# Evolutionary conservation of dual Sec translocases in the cyanelles of *Cyanophora paradoxa*

**DOI:** 10.1186/1471-2148-8-304

**Published:** 2008-11-01

**Authors:** Fumie Yusa, Jürgen M Steiner, Wolfgang Löffelhardt

**Affiliations:** 1 SLT, Nagahama-city, Shiga-ken 526-0829, Japan; 2 Max F. Perutz Laboratories, University of Vienna, Department of Biochemistry, Dr. Bohrgasse 9, A-1030 Vienna, Austria

## Abstract

**Background:**

Cyanelles, the peptidoglycan-armored plastids of glaucocystophytes, occupy a unique bridge position in between free-living cyanobacteria and chloroplasts. In some respects they side with cyanobacteria whereas other features are clearly shared with chloroplasts. The Sec translocase, an example for "conservative sorting" in the course of evolution, is found in the plasma membrane of all prokaryotes, in the thylakoid membrane of chloroplasts and in both these membrane types of cyanobacteria.

**Results:**

In this paper we present evidence for a dual location of the Sec translocon in the thylakoid as well as inner envelope membranes of the cyanelles from *Cyanophora paradoxa*, i. e. conservative sorting *sensu stricto*. The prerequisite was the generation of specific antisera directed against cyanelle SecY that allowed immunodetection of the protein on SDS gels from both membrane types separated by sucrose density gradient floatation centrifugation. Immunoblotting of blue-native gels yielded positive but differential results for both the thylakoid and envelope Sec complexes, respectively. In addition, heterologous antisera directed against components of the Toc/Tic translocons and binding of a labeled precursor protein were used to discriminate between inner and outer envelope membranes.

**Conclusion:**

The envelope translocase can be envisaged as a prokaryotic feature missing in higher plant chloroplasts but retained in cyanelles, likely for protein transport to the periplasm. Candidate passengers are cytochrome *c*_6 _and enzymes of peptidoglycan metabolism. The minimal set of subunits of the Toc/Tic translocase of a primitive plastid is proposed.

## Background

The Sec translocase which provides a major pathway of protein translocation from the cytosol across the cytoplasmic membrane in bacteria is best investigated in *Escherichia coli*. The core of the translocase consists of SecY, SecE, and SecG [1]. This SecYEG complex and the hydrophilic protein SecA, which acts as the translocation ATPase, are the primary components of the Sec translocation machinery. During the integration step of membrane proteins, YidC (a bacterial homolog of mitochondrial Oxa1 and chloroplast Alb3) is required, eventually in addition to the Sec translocase [2,3]. The Sec tranlocation machinery is unable to translocate folded proteins (in contrast to the Tat machinery). Therefore, a Sec-specific chaperone, e.g. SecB, binds to candidate proteins preventing them to complete folding prior to export.

The Sec translocase is one of the examples of "conservative sorting", i.e. the retainment of prokaryotic preprotein translocases in the membranes of endosymbiont-derived organelles. Cyanobacteria, the ancestors of chloroplasts are prokaryotes with an endomembrane system in addition to the envelope membrane(s). Little is known about the Sec translocase of cyanobacteria, other than the analogous composition of the core: SecY (product of a single gene), SecE, and SecG as deduced from the sequences deposited in CyanoBase http://plant1.kazusa.or.jp:3010/. Here, export from the cytosol could occur either into the periplasm or into the thylakoid lumen. Indeed, a dual localization of SecY protein in both cytoplasmic and thylakoid membranes from the cyanobacterium *Synechococcus *PCC7942 was reported [4]. Periplasmic proteins as well as lumenal proteins of cyanobacteria are synthesized as larger precursors containing N-terminal signal sequences which show subtle differences depending on the localization of the mature proteins [5]. On the other hand, there is no indication for a Sec translocase in inner envelope membranes of chloroplasts, but it is well documented in thylakoid membranes of higher plants [6-8]. In contrast to cyanobacteria, there is no proof for the existence of the SecG subunit, i.e. no hit in the *Arabidopsis *genome. However, primitive plastids (rhodoplasts and secondary plastids of the red line) harbor a *ycf47 *on their genomes thought to be an equivalent of *secG *[9]. The soluble SecA subunit in chloroplasts has also been characterized [10,11]. Chloroplast SecY forms a complex with SecE of approximately 180 kDa in *Arabidopsis thaliana *[7]. Using different methods, 120 kDa and 160 kDa Sec complexes were detected in thylakoid membranes from *A. thaliana *[12]. The thylakoid membrane protein Alb3 was reported to associate with the chloroplast Sec-translocase in *A. thaliana*: The difference in molecular weight between the 120 kDa and 160 kDa Sec translocases accounts for the presence or absence of Alb3 in the Sec complex [12].

To follow the pathway, how chloroplasts modified the Sec translocon during evolution, primitive plastids are promising. Cyanelles (muroplasts) are plastids surrounded by a peptidoglycan wall, only encountered in glaucocystophyte algae. Glaucocystophyte algae are thought to represent primordial phototrophic eukaryotes, branching off after the single primary endosymbiotic event [13,14]. The sequence of SecY from *Cyanophora paradoxa *is already known [15]. The cyanelle gene was shown to complement the thermosensitive *secY24 *mutation [16] in *E. coli*. There is no evidence for an additional, nuclear-encoded SecY in *C. paradoxa*. In contrast to chloroplasts, cyanelles have a defined periplasmic space between their envelope membranes, harboring the peptidoglycan wall, eight penicillin-binding proteins and a number of additional enzymes involved in peptidoglycan modification and degradation [14]. The intra-cyanellar localization of cyanelle cytochrome *c*_6 _was determined [17] indicating translocation of this protein to the periplasm as well as to the thylakoid lumen. The operation of a Sec translocon at the thylakoid membrane of cyanelles was recently demonstrated [18] whereas a possible localization at the inner envelope membrane was still an open question. Here we present evidence for dual Sec translocases in inner envelope membranes as well as thylakoid membranes, another feature shared between cyanobacteria and cyanelles, but not chloroplasts. Chloroplast inner envelope membranes contain the Tic component of the protein import system of the envelope, whereas the outer membranes contain the Toc component [19]. Cyanelles harbor a functional equivalent of the Toc/Tic machinery in their envelope [20] and we tried to assign individual components to the inner and outer membranes, respectively.

## Methods

### Growth, cyanelle, and membrane isolation

*Cyanophora paradoxa *LB555UTEX was grown as previously described [21]. Intact cyanelles were isolated as described in [17]. Intact cyanelles were washed with 50 mM Hepes buffer, pH 7.5, 1 mM EDTA, 2 mM EGTA, 1 mM MgCl_2_(HEM) and 0.5 M sucrose and pelleted at 2,400 rpm, 2 min again (Beckman Sorvall SS34 rotor). The pellet was resuspended in HEM buffer with 0.6 M sucrose and treated with 0.1 mg/ml lysozyme at room temperature for 1 hr in the dark to digest the peptidoglycan wall. The cyanelles were then broken by Beads Beater (FP120), 5 × 15 sec. The homogenate was centrifuged at 5,000 × g for 10 min at 4°C to remove unbroken cyanelles, and the chlorophyll concentration of supernatant was adjusted to 0.5 mg/ml by adding HEM buffer with 0.6 M sucrose. The sucrose concentration of the homogenate was adjusted to 52%(w/v) by adding 80%(w/v) sucrose. For separation of envelope membranes by sucrose density floatation centrifugation, the following sucrose gradient was built in a 38 ml-centrifuge tube: 4 ml of 60%(w/v) sucrose containing HEM buffer and 4 ml of the sample was placed successively from the bottom. 3 ml of 45%(w/v), 12.5 ml of 39%(w/v), 12.5 ml of 15%(w/v) sucrose in HEM buffer and 1 ml of HEM buffer were layered on the sample. Centrifugation was performed at 122,000 × g for 16 h (Beckman L-80 Ultracentrifuge, SW 28) at 4°C. Thylakoid membranes appeared in the 45% layer and the inner envelope membrane band was found between 39% and 15% sucrose. Each membranes were pelleted by centrifugation. The purity of the preparations was checked by absorption spectra and pigment analysis via HPLC [22,23].

### Gel electrophoresis

In general SDS-polyacrylamide gel electrophoresis of proteins under denaturing conditions was carried out according to Laemmli [24]. To account for the reported thermosensitivity of SecY, treatment with sample buffer was done at 37°C for 30 min or 60°C for 10 min. Blue Native(BN)-PAGE was performed as described elsewhere [25,26], and SDS-PAGE as second dimension was carried out as described in [27]. BN-PAGE of intact cyanelles (0.5 mg Chl *a*) involved 30 min treatment in 375 ml lysis buffer with detergent (1.67% digitonin) at 4°C and removal of the cyanelles by centrifugation for 30 min at 18,000 × *g*.

### Western blotting

After separation of proteins by SDS-PAGE or BN-PAGE, Western blotting was performed. The transfer was done in a BioRad Trans-Blot Cell for 16 h at 4°C at a constant voltage of 25V. To check transfer of proteins the membrane was reversibly stained with Ponceau Red S at room temperature, washed 2–3× with TBST (25 mM Tris pH 7.4, 150 mM NaCl and 0.1% Tween 20), and unspecific binding sites were blocked by incubation in 10% milk powder in TBST or 3% BSA in 25 mM Tris, pH 7.4, 150 mM NaCl for 2 or 3 h. After three washings in TBST (15 min and 5 min × 2) the membrane was incubated with the primary antibody for 1 h, washed in TBST (15 min and 5 min × 2), and incubated for 1 h with the corresponding secondary antibody goat anti-rabbit IgG/alkaline phosphatase (AP) (Promega) for antisera diluted 1:7500 in TBST or anti rabbit IgG/horseradish peroxidase (Pharmacia) for antisera diluted 1:10000 in TBST. (As a rule, AP was used for SDS-PAGE, horseradish peroxidase for BN-PAGE) In case of AP, after washing in TBST (15 min and 5 min × 4) and, briefly, in AP buffer, the membrane was incubated in 5 ml AP buffer containing 33 μl NBT (50 mg/ml, in 70% DMF) and 16.5 μl BCIP (50 mg/ml, in 100% DMF) until bands were visible. The reaction was stopped by washing with H_2_O. In case of horseradish peroxidase, after washing 3× with TBST (15 min each), the membrane was stained with a chemiluminescent reagent (Supersignal, Pierce, Rockford, IL), and exposed to X-ray film.

### Affinity purification of antisera directed against cyanelle SecY

A peptide consisting of the 17 C-terminal amino acids of cyanelle Sec Y plus an added cystein, Cys-KREPLKRDFSKRRSAN for coupling to keyhole limpet hemocyanin was used for the immunization of rabbits (Gramsch Labs., Schwabhausen, FRG). The antisera were tested for reaction with dilutions of the peptide and by western blotting of cyanelle thylakoid membrane proteins. Some bands in the appropriate size region were obtained but the signal was weak and the background was strong. To render the signal more specific and to reduce the background, the antisera were affinity-purified on a column containing immobilized peptide. The affinity-purified antisera were again tested for reaction with dilutions of the peptide and by western blotting of thylakoid membrane proteins from cyanelles.

Peptide coupling using Thiopropyl Sepharose 6B: 1 g Thiopropyl Sepharose 6B (Amersham-Pharmacia) was washed twice with ddH2O and twice with coupling buffer (0.1 M Tris, 0.5 M NaCl, 1 mM EDTA, pH 7.5) in a 50 ml Falcon tube carefully avoiding to produce air bubbles. After decanting the supernatant, 10 ml coupling buffer was added. The suspension was poured into a column (diameter 1.2 cm) to give a bed length of 5 cm. 10 ml coupling buffer including 10 mg peptide were added at a speed of 5 ml/h. Subsequently, the column was washed with 10 ml of 20 mM sodium acetate buffer, ph 4.5 and then with 10 ml sodium acetate buffer including 17 mM mercaptoethanol (about 5 ml/h). The effluent was collected in 1 ml fractions and measured photometrically at 343 nm. Finally, 40 ml sodium acetate buffer was applied to the column, at first slowly then increasingly faster till 30 ml/h were reached. The column was then equilibrated with 10 ml peptide buffer (100 mM sodium phosphate, 200 mM NaCl, 1 mM EDTA, pH 6.0).

Affinity chromatography: First, 10 ml peptide buffer was run into the column with 30 ml/h. Then sterile-filtrated antiserum (1:1 dilution with peptide buffer) was loaded onto the column at 20 ml/h. The flow-through was collected. After that, 5 × 50 ml peptide buffer were used to wash the column, gradually increasing the flow rate to 60 ml/h. All effluents were collected and protein content was determined with Bradford reagent (BioRad). After all washing steps were finished and no more protein was detectable in the effluents, 50 ml elution buffer (100 mM glycine, 150 mM NaCl, pH 2.45) was run into the column at a speed of 30 ml/h. 1 ml fractions of eluate were collected in Eppendorf vials containing 100 μl neutralization buffer (3 M Tris/HCl, pH 7.8) and mixed immediately. Then 50 μl aliquots were taken for protein determination. These 50 vials were ranked as -, +, 2+, 3+, according to the intensity of the color. 3+ vials were combined to Pool A and 5 M NaCl was added to final concentration of 250 mM. 2+ vials were combined to Pool B, and + vials were combined to Pool C. After that, dialysis was performed for 3 × 12 h against 1× PBS, 1 mM EDTA, pH 8.0, and the samples were centrifuged (3000 × *g *at 4°C for 10 min). 2% sodium azide (0.01 volumes) was added to the supernatants and the affinity-purified antisera were stored at 4°C.

## Results and discussion

### Dual location of the Sec preprotein translocase in the cyanelles

**Figure 1:**
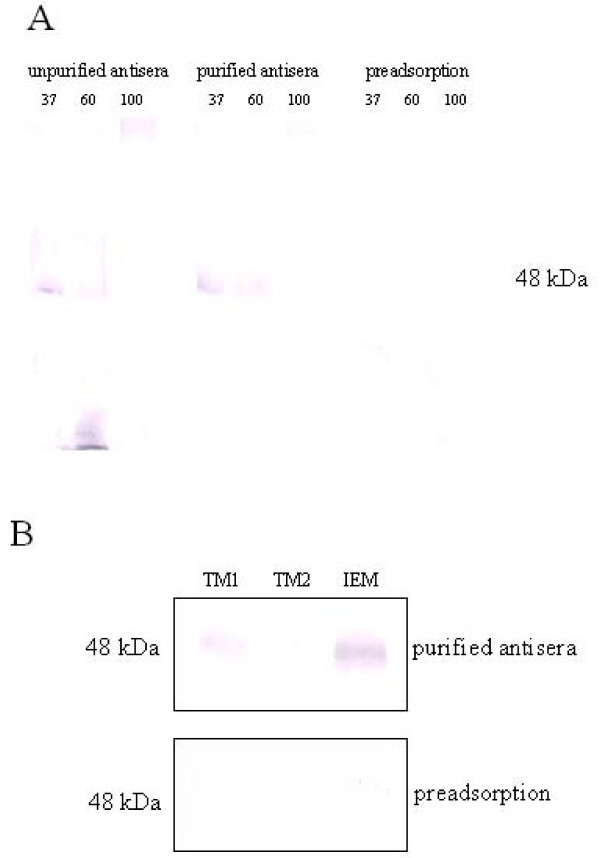
**Western blotting with SecY antisera of cyanelle membranes separated on SDS gels. **Preadsorption, detection with affinity purified antisera which underwent preadsorption to the heptadekapeptide; TM, thylakoid membranes; IEM, inner envelope membranes. (A) Western blotting of thylakoid membranes (30 μg protein) with unpurified vs. affinity purified cyanelle SecY antisera. Thylakoid membranes were treated with sample buffer at 37°C for 30 min (37), 60°C for 10 min (60), 100°C for 3 min (100). (B)Western blotting of cyanelle membranes separated on SDS gels with affinity purified anti-cyanelle SecY. TM1 (20 μg protein) and IEM (30 μg protein) were treated with sample buffer at 37°C for 30 min, TM2 (20 μg protein) was treated at 100°C for 3 min.

When we postulate conservative sorting in analogy to cyanobacteria, the cyanelle Sec translocases must reside in both thylakoid and inner envelope membranes. Recently, a method for separation of cyanelle membranes was established [23]. To confirm this hypothesis, it was mandatory that thylakoid membranes and inner envelope membranes were subjected to western blotting with antisera directed against constituents of the Sec translocase of cyanelles. Antisera were raised against the C-terminal hexadecapeptide of cyanelle SecY plus an added cysteine for coupling of the hapten to keyhole limpet hemocyanin. To account for the reported thermosensitivity of SecY [28], the thylakoid membranes were treated with sample buffer at 37°C for 30 min, 60°C for 10 min, and 100°C for 3 min. The preimmune sera and all antisera were checked for presence of antibodies directed against the hapten by reaction with dilutions of the antigenic peptide. Strong signals were obtained with the antisera, whereas the preimmune sera showed only weak reaction (data not shown). A band around 50 kDa was decorated by the antisera on thylakoid protein blots (Fig. 1A). The calculated molecular weight of cyanelle SecY is 53 kDa but the apparent MW on SDS gels is lower due to its high hydrophobicity [15]. Since there was strong background, affinity purification was required. The affinity-purified antisera against SecY of cyanelles were again tested by western blotting of cyanelle thylakoid membrane proteins and the signals became stronger with much less background (Fig. 1A). The signal appeared as a band of approximately 48 kDa that vanished upon treatment at 100°C and was reduced at 60°C. This 48 kDa band was concluded to represent SecY: i) the size corresponded well to the calculated molecular weight of 53 kDa (when the highly hydrophobic character of this protein is taken into account), ii) this signal disappeared upon heating and iii) upon preadsorption (Fig. 1A).

Western blotting of cyanelle inner envelope membrane proteins was performed and a 48 kDa band was also decorated with cyanelle-SecY antisera (Fig. 1B). The intensity of the signal excluded its origin from thylakoid membrane contamination, which was less than 3%. This signal also vanished after preadsorption (Fig. 1B). Hence we propose that inner envelope membranes also contain a SecY translocon, as was previously observed in the cyanobacterium, *Synechococcus *PCC7942 [4]. In our belief, this provides an even more pronounced example of conservative sorting than that found in higher plants, i.e. the retention of both cyanobacterial Sec translocases in plastid membranes.

### The Sec complex in cyanelle thylakoid membranes

**Figure 2:**
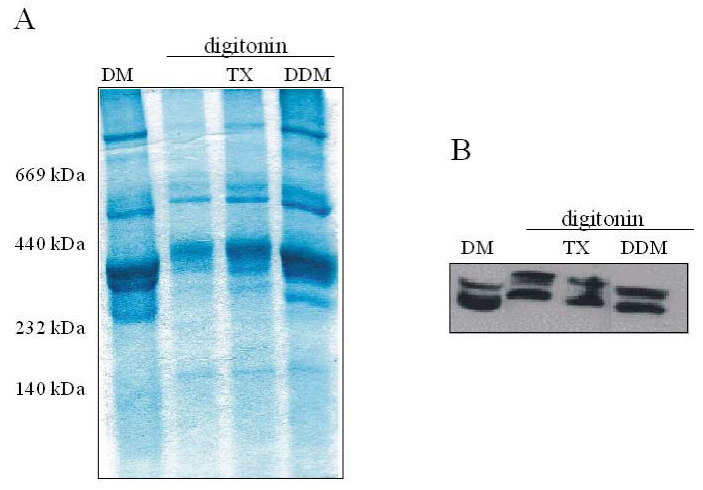
**Analysis of protein complex on thylakoid membranes of cyanelles by BN-PAGE and the corresponding western blot against cyanelle-SecY**. DM, decyl maltoside; DDM, dodecyl maltoside; TX, Triton X-100 (A) purified thylakoid membranes (30 μg of chlorophyll *a*) were solubilized with 2% decyl maltoside, 1.67% digitonin and combination of 1.67% digitonin and 0.03% Triton X-100 or 0.5% dodecyl maltoside. (B) Western blotting against cyanelle-SecY.

SecY is known as a member of the SecYE(G) complex. BN-PAGE is a suitable method for investigating the size of the cyanelle Sec complex. The thylakoid membranes were either treated by decyl maltoside or digitonin. A combination of digitonin with dodecyl maltoside or Triton X-100 was also used. The membrane protein complexes were clearly separated by BN-PAGE (Fig. 2A). Upon solubilization with digitonin, several bands became apparent: 770 kDa, 700 kDa, 640 kDa, 600 kDa, 550 kDa, 420 kDa, 390, 340, 280, 160. Most bands were shifted downward by approximately 20 kDa when dodecyl maltoside (in addition to digitonin) or decyl maltoside were used. The gel was tested by western blotting with cyanelle-SecY antibody (Fig. 2B). 340 kDa and 300 kDa bands were decorated upon digitonin treatment, 320 kDa and 280 kDa bands when decyl maltoside or (in addition) dodecyl maltoside were used. We consider these 340 kDa (320 kDa) and 300 kDa (280 kDa) bands as Sec complexes, their size difference being independent of the detergent mixture used. The presence or absence of an Alb3-like protein was reported to shift the size of higher plant thylakoid membrane Sec complexes by about 40 kDa [12]. The 300 kDa (280 kDa) band was decorated stronger than the 340 kDa (320 kDa) band: thus the 300 kDa (280 kDa) band should represent the major Sec complex in cyanelle thylakoid membranes. After BN-PAGE, SDS-PAGE was performed as second dimension (not shown). Upon western blotting, both 48 kDa protein spots arising from the 280 kDa and 320 kDa bands were decorated, the former more strongly. This also corroborated that the 280 kDa band corresponds to the major Sec complex in cyanelle thylakoid membranes (Fig. 2). Future progress in *Cyanophora *genomics might allow in combination with proteomics to identify also SecE and SecG. The importance of SecG and its topology inversion for SecA-dependent protein translocation was recently re-emphasized [29].

### The Sec complex in cyanelle inner envelope membranes

To investigate a potential localization of the Sec complex in cyanelle inner envelope membranes, again BN-PAGE was performed. To get a higher yield of inner envelope membranes, cells grown for 3 weeks were used [23] (normally, they were 1~2 weeks old). Since decyl maltoside has been successfully used to isolate the chloroplastic Tic translocon and also has been chosen for solubilization of inner envelope membranes for BN-PAGE [30], inner envelope membranes of cyanelles were treated with decyl maltoside. Thylakoid membranes from 3 weeks cells were done in parallel. A number of protein complexes were separated from cyanelle inner envelope membranes by BN-PAGE (not shown) but the resolution was not as good as shown for thylakoid membranes (Fig. 2A.). The reason for that could be the age of the cells. However, western blotting with anti-SecY decorated only one distinct band at the position (or slightly lower) of the 280 kDa thylakoid band. We propose that this band represents the Sec complex of inner envelope membranes. Although less total protein was used, the signal from inner envelope membrane BN-PAGE was significant, excluding thylakoid membrane contamination as a potential source. This dualism reflects the need for protein translocation from the stroma not only to the thylakoid lumen but also to the periplasm of cyanelles. The (partially) soluble component of the translocase, SecA, acting in the cyanelle stroma, could provide the necessary energy input for both translocation processes since the required digalactosyldiacylglycerol is a constituent of thylakoid as well as inner envelope membranes [31]. We found a 700 bp EST in our library (GenBank EC664402) with significant sequence similarity to SecA proteins from cyanobacteria and plastids. The genes for the other membrane-bound components of the Sec translocon are not known for *C. paradoxa *where the presently available EST's cover about 30% of the estimated total of 12000 genes: *ycf47 *(*secG*) does not reside on the plastome in contrast to red algae and *secE *has not been found yet either.

Dual localization in thylakoid and inner envelope membranes has also been reported for S-Adenosyl-L-methionine: Mg-protoporphyrin IX methyltransferase from *Arabidopsis thaliana*, though in this case the association seems to be confined to one leaflet of the respective membranes [32].

### Correlation to the cyanelle protein import apparatus

**Figure 3:**
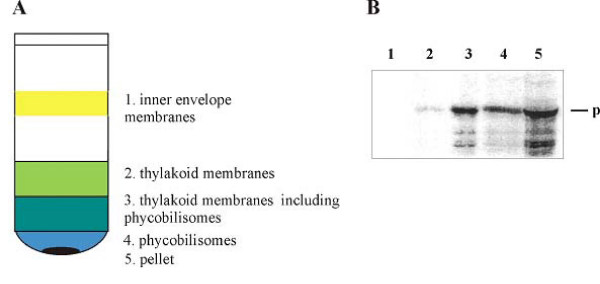
**A. Fractions obtained from cyanelle lysate by sucrose density gradient floatation centrifugation. ** B. Binding of ^35^S-labeled pre-FNR from C. paradoxa to isolated cyanelles under non-import conditions, lysis and fractionation. Aliquots of the fractions (40% of 1, 5% of 2, 5% of 3, 30% of 4 and 50% of 5) were precipitated with TCA and run on a SDS gel. Radioactivity in the pellet (fraction 5) is likely bound to yet intact cyanelles. The band at 35 kDa represents a degradation product [42] rather than processed mature protein.

**Figure 4:**
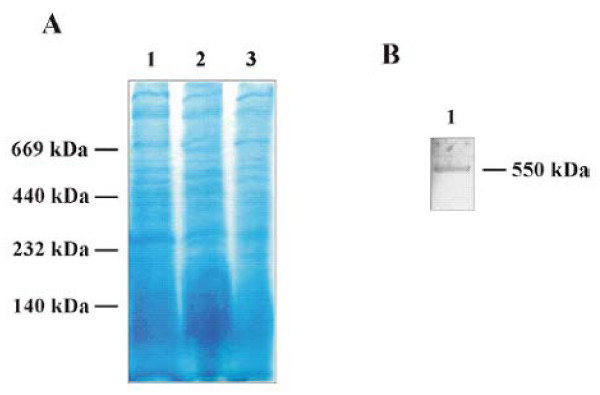
**Separation of protein complexes from the OEM and periplasm of cyanelles by BN-PAGE applied under gentle (hypertonic) conditions where no noticeable lysis of IEM and liberation of stromal proteins occurred**. A. Intact, import-competent cyanelles (500 μg chlorophyll) were treated with (1) 1.67% digitonin, (2) 1.67% digitonin and 0.5% dodecyl maltoside and (3) 1.67% digitonin and 0.03% Triton X-100. B. Western blot of lane 1 (αToc75-III).

**Figure 5:**
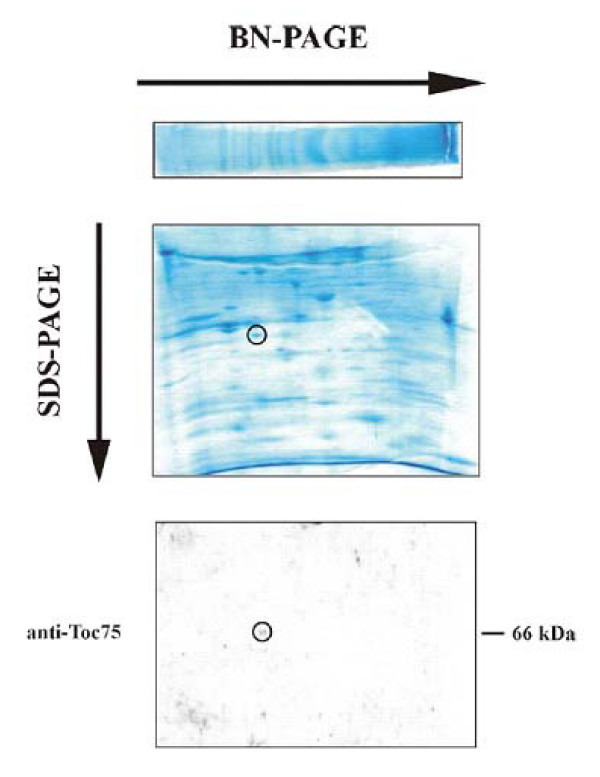
Analysis of the protein complexes obtained via BN-PAGE (Fig. 5) by 2^nd ^dimension SDS-PAGE on a 10% gel and western blotting (αToc75-III).

**Figure 6:**
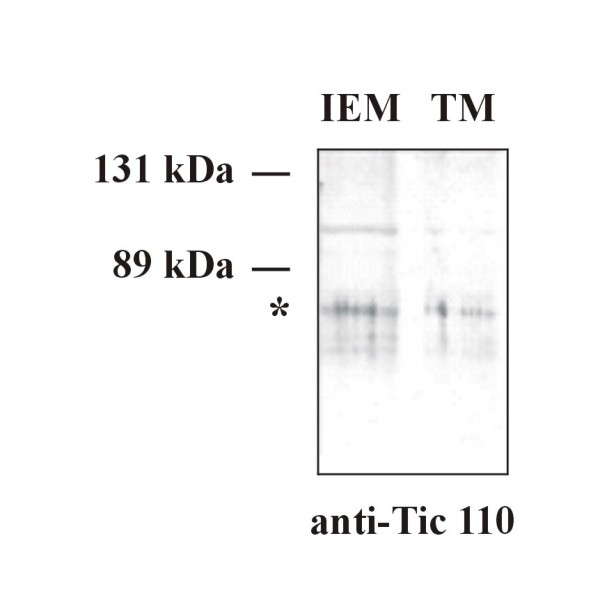
**Western blots (αTic110) of cyanelle IEM (20 μg protein, A) and thylakoid (30 μg protein, B) proteins separated by SDS-PAGE on a 10% gel**. *Degradation product or cross-reacting protein.

In contrast to chloroplasts [33] and cyanobacteria [34], the isolation of cyanelle outer envelope membranes (OEM) is a difficult task due to the influence of (partially) degraded peptidoglycan on the buoyant density [23] and due to the absence of suitable marker proteins. Another problem is partial loss of outer membranes during cyanelle isolation [35]. Accordingly, upon heterologous western blots using antisera raised against Toc75 from *Synechocystis *sp. PCC6803, the best result was obtained when whole *Cyanophora *was used for the SDS gels [20]. Therefore, binding of a labeled precursor to the Toc translocon of intact (import-competent) isolated cyanelles under conditions preventing translocation (0°C, dark) was used to identify OEM. After lysozyme treatment and mechanical lysis of cyanelles and sucrose density gradient floatation centrifugation, largely 70% of the radioactivity of ^35^S-labeled pre-ferredoxin-NADP^+ ^oxidoreductase from *C. paradoxa *coincided with the intact, i. e. phycobilisome-bearing thylakoid membranes (Fig. 3). The remainder was distributed between the phycobilisome layer and the pellet. A distribution of OEM between thylakoids and phycobilisomes with a shift towards the latter upon prolonged (40 h) centrifugation was recently reported. The OEM preparations obtained [23] were subjected to several cycles of sedimentation and detergent extractions, conditions that might interfere with the biological activity. In order to circumvent these difficulties, we prepared intact cyanelles as described above and carefully solubilized outer membrane and periplasmic protein complexes according to the principles of blue-native gel electrophoresis [25,26], a method not amenable to chloroplasts. The advantage of the stabilizing peptidoglycan wall is that during short interaction with non-ionic detergent the inner envelope membrane as well as the stroma content and the thylakoids are largely left intact: after removal of the "extracted" cyanelles by centrifugation only very small amounts of phycocyanin were found in the supernatant used for BN-PAGE. A well-defined pattern of bands was obtained (Fig. 4A). Antisera against Toc75-III from *A. thaliana *decorated a band of approximately 550 kDa (Fig. 4B), which is close to the size reported for the Toc complex from higher plants [33] though the subunit composition most likely is different. No signal was obtained with αTic110 under these conditions. When the BN gel was subjected to SDS-PAGE in the second dimension, a spot at approximately 70 kDa produced a signal upon reaction with αToc75-III (Fig. 5). Cyanelle inner envelope membranes (IEM) prepared by conventional sucrose density gradient centrifugation that were compentent for late steps in peptidoglycan biosynthesis [36] yielded a signal at approximately 50 kDa upon western blotting of SDS gels with αTic55 from *Synechocystis *sp. PCC6803, whereas antibodies raised against the pea protein were ineffective [20]. In order to probe further Tic components, cyanelle IEM was prepared *via *sucrose density gradient floatation centrifugation and subjected to western blotting with αTic110 and αTic40, both from *A. thaliana*. A signal in the expected size range was obtained with αTic110 only (Fig. 6). A thylakoid membrane control showed this band only faintly, which we ascribe to a slight (unavoidable) contamination with IEM. This was corroborated by an EST from *C. paradoxa *with sequence similarity to Tic110 (GenBank EC662367). When IEM was prepared in this way, no signals were obtained with αToc75. Also, the very low amount of chlorophyll in the IEM fraction indicated that contamination of IEM with both thylakoids and OEM was negligible. The ratio zeaxanthin:chlorophyll *a *was 18:1 for IEM and 1:14 for TM [22,23]. When IEM was solubilized with 2% dodecyl maltoside the antibody reaction with αTic110 did not work on BN gel blots prohibiting a size estimation of the cyanelle Tic complex. From these preliminary data, the minimal composition of the envelope translocon for import of cytosolic precursors would be Toc75-Tic110 as predicted [37,38].

## Conclusion

The passenger proteins for the Sec translocase in the inner envelope membrane seem to be nucleus-encoded in *C. paradoxa*: There is no candidate gene on the cyanelle genome [14] with a product potentially residing in the periplasm and displaying a signal peptide in the precursor form. Therefore we cannot compare potential differences in the signal peptides of lumen- and periplasm-sorted proteins as was done for cyanobacteria [5]. The results obtained with the nuclear-encoded cytochrome *c*_6 _[17] indicate the possibility of dual sorting directed by the same signal sequence.

The Tat translocase responsible for folded and bulky precursors thus far was (indirectly) localized to cyanobacterial inner envelope membranes only [39]. However, cyanobacterial Rieske Fe/S protein with its uncleaved (twin-arginine) signal anchor peptide and a few thylakoid lumen proteins [40] are also candidate passengers for a Tat translocase in the thylakoid membrane. In chloroplasts, the Tat translocase is thought to be confined to the thylakoid membrane [8]. For cyanelles, there is indirect evidence for a Tat translocase in the thylakoid membrane [18]. Some Tat passengers involved in peptidoglycan biosynthesis http://plant1.kazusa.or.jp:3010/ might necessitate a dual location of the Tat translocase, too. At present this is a mere speculation, though "fully" conservative sorting of unfolded and folded precursors in cyanelles would seem an appealing idea, in line with the unique bridge position these peculiar plastids occupy.

Identification of cyanelle translocon components is hampered by the low expression rate and therefore low representation in existing EST libraries, in contrast to the situation in higher plant chloroplasts [41].

## Authors' contributions

FY performed cyanelle membrane isolation, pigment analysis, affinity purification of antisera, electrophoreses and western blotting. JMS participated in the design of the study and performed the labeled precursor experiments. WL conceived of the study and wrote the manuscript which was read and approved by all authors.
